# Faecal pharmacokinetics, microbiome, and bile acid changes in healthy subjects given intravenous followed by oral omadacycline; a Phase 1 clinical trial

**DOI:** 10.1093/jac/dkaf278

**Published:** 2025-08-12

**Authors:** Jinhee Jo, Travis J Carlson, Chenlin Hu, John C Williamson, Yolanda T Belin, Thomas D Horvath, Sigmund J Haidacher, Eugénie Bassères, Khurshida Begum, M Jahangir Alam, Kevin W Garey

**Affiliations:** Department of Pharmacy Practice and Translational Research, University of Houston College of Pharmacy, Houston, TX, USA; Division of Pharmacotherapy and Translational Sciences, The University of Texas at Austin College of Pharmacy, San Antonio, TX, USA; Pharmacotherapy Education and Research Center, The University of Texas Health Science Center at San Antonio, San Antonio, TX, USA; Department of Pharmacotherapy and Pharmacy Services, University Hospital, San Antonio, TX, USA; Department of Pharmacy Practice and Translational Research, University of Houston College of Pharmacy, Houston, TX, USA; Department of Internal Medicine, Section of Infectious Diseases, Wake Forest University School of Medicine, Winston-Salem, NC, USA; Department of Internal Medicine, Section of Infectious Diseases, Wake Forest University School of Medicine, Winston-Salem, NC, USA; Department of Pathology and Immunology, Baylor College of Medicine; Houston, TX, USA; Texas Children’s Microbiome Center, Department of Pathology, Texas Children’s Hospital, Houston, TX, USA; Department of Pathology and Immunology, Baylor College of Medicine; Houston, TX, USA; Texas Children’s Microbiome Center, Department of Pathology, Texas Children’s Hospital, Houston, TX, USA; Department of Pharmacy Practice and Translational Research, University of Houston College of Pharmacy, Houston, TX, USA; Department of Pharmacy Practice and Translational Research, University of Houston College of Pharmacy, Houston, TX, USA; Department of Pharmacy Practice and Translational Research, University of Houston College of Pharmacy, Houston, TX, USA; Department of Pharmacy Practice and Translational Research, University of Houston College of Pharmacy, Houston, TX, USA

## Abstract

**Background:**

There is an urgent need to develop new antimicrobials effective intravenously for *Clostridioides difficile* infection (CDI). Omadacycline is an aminomethylcycline tetracycline available orally and intravenously with potent *in vitro* activity against *C. difficile* and a low propensity to cause CDI. The purpose of this study was to assess the safety, faecal pharmacokinetics, microbiome and bile acid changes in healthy subjects given a course of intravenous omadacycline with oral omadacycline step down after 5 days.

**Methods:**

This Phase 1, open-label study was conducted in healthy volunteers 18–40 years. Subjects received a 5-day course of omadacycline given intravenously followed by 5 days of oral omadacycline. Stool samples were analysed for omadacycline concentrations, gut microbiome changes and bile acid changes from baseline.

**Results:**

Eight healthy volunteers aged 30 ± 4 years (50% Female) were recruited and all completed therapy. All subjects had detectable omadacycline stool concentrations after 48 hours of intravenous dosing and averaged 195 ± 97 µg/g (mean ± SD) by day 5. Omadacycline concentrations increased rapidly after the start of oral therapy on day 6 with average concentrations of 854 ± 404 µg/g of stool by day 10. Microbiome and bile acid evaluations showed preservation of key microbiome taxa that confer health benefit and preservation of bile acid homeostasis.

**Conclusion:**

Intravenous omadacycline followed by oral step-down administration in healthy adults achieved high faecal concentrations while preserving key bacterial species and bile acid homeostasis in the gut. These findings support Phase 2 studies directed towards the development of omadacycline as a CDI-targeted antibiotic.

## Introduction

*Clostridioides difficile* infection (CDI) is a common healthcare-associated infection accounting for ∼500 000 cases and 29 000 deaths annually in the USA.^1,2^ Current treatment guidelines recommend one of two antibiotics for initial treatment of CDI with fidaxomicin preferred due to a lower CDI recurrence rate but oral vancomycin also recommended as an alternate. Both agents can only be given orally.^3,4^ Oral metronidazole was recommended historically but is no longer guideline-recommended to be given as initial therapy due to decreased clinical response rates, high recurrence likelihood and increased antimicrobial resistance.^5,6^ Intravenous antibiotic therapy is recommended only for fulminant CDI (FCDI). FCDI occurs in 3%–5% of cases and is associated with a high mortality rate of 30%–40% due to a high likelihood of the ileus preventing oral antibiotics from passing to the colon, the site of infection for CDI.^7^ Despite the inferior success rate with oral metronidazole and emerging resistance, metronidazole given intravenously is guideline-recommended for FCDI along with vancomycin given orally or by rectum due to the lack of other CDI-directed intravenous antibiotics.^8^ Thus, there is an urgent need to develop new intravenous antimicrobials effective for the treatment of CDI.

CDI is caused by *C. difficile* after perturbation of the gut microbiome, most commonly by prior use of high-risk antibiotics, and is characterized by decreased proportions of the bacterial phyla Bacillota (formerly Firmicutes), Bacteroidota (formerly Bacteroidetes) and Actinomycetota (formerly Actinobacteria) with subsequent overgrowth of Pseudomonadota (formerly Proteobacteria).^9^ These changes increase propensity for CDI by eliminating taxa responsible for bile acid metabolism, leading to higher concentrations of primary bile acids that promote *C. difficile* spore germination. Intravenous antibiotics in development for the treatment of CDI should have high colonic concentrations, exhibit potent activity against *C. difficile* strains, but lack activity against healthy gut microbiota that confers CDI colonization resistance and maintains bile acid homeostasis. Pharmacokinetic and microbiome effects can be investigated in early clinical studies including healthy volunteer studies allowing early recognition of potentially valuable new therapies.^10^

Omadacycline is an aminomethylcycline antibiotic available in both oral and intravenous formulations. It is approved by the US Food and Drug Administration for community acquired pneumonia and acute bacterial skin and skin structure infections in adults. Omadacycline has potent *in vitro* activity against *C. difficile*, including the epidemic 027 ribotype, and clinical trials have shown low propensity for associated CDI.^11–15^ In a recent Phase 1 healthy volunteer study, oral omadacycline achieved high faecal concentrations that exceeded the minimum inhibitory concentration (MIC) for *C. difficile* while preserving key bacterial species in the gut microbiome.^16^ However, this study was limited to the use of oral omadacycline. The purpose of this study was to assess the faecal pharmacokinetics, microbiome, and bile acid changes in healthy subjects given a course of intravenous omadacycline with oral step down after 5 days.

## Materials and methods

### Study design and participants

This Phase 1 open-label study was conducted at the Infectious Diseases Clinical Trials Center of Atrium Health Wake Forest Baptist Medical Center (NCT05515562). Subjects were recruited from March 2023 to July 2023. The study was approved by the Institutional Review Board of Wake Forest University Health Sciences (IRB00081259) and was conducted in accordance with International Council for Harmonization Good Clinical Practice guidance. Written informed consent was obtained from all participants before performing any study procedures. Eight subjects were initially planned for enrolment.

### Participants

Male or female (self-reported) participants were eligible to enrol if they met the following criteria: (i) age between 18 and 40 years, and (ii) considered healthy with no significant medical history, such as diabetes; obesity; cardiovascular, gastrointestinal, hepatic or renal disease (among others) and current tobacco use. Exclusion criteria included known hypersensitivity to omadacycline or tetracycline-class antibiotics, receipt of an antibiotic within 90 days prior to enrolment, consumption of a probiotic within 30 days prior to enrolment, pregnancy, or breastfeeding. Participants who were female of child-bearing potential received a urine pregnancy test during screening and again on day 5 to confirm non-pregnant status. Volunteers who were excluded due to probiotic or antibiotic consumption were allowed to re-screen later.

### Procedures

Subjects received a 10-day course of omadacycline that consisted of 200 mg given intravenously on day 1, followed by 100 mg given intravenously daily on days 2–5 and then omadacycyline 300 mg given by mouth daily on days 6–10. Participants were instructed to take oral doses on an empty stomach (no food within 4 hours before to 2 hours after each dose). All intravenous omadacycline doses were administered under direct observation. Participants recorded the date and time of stool specimens and oral doses in a diary, which was reviewed by the research team at each visit. Stool samples from each participant were collected before dosing at the screening visit within 5 days before study day 1, daily (if possible) during therapy on days 1–10 and at two follow-up visits [day 13(+1) and day 31(±1)]. Stool specimens collected at the Clinical Trials Center were processed immediately following collection. Participants who generated a stool sample at other times were asked to deliver the sample to the Clinical Trials Center on the same day up to 4:30 pm. Samples generated after hours were kept refrigerated by the participant until delivery the next day. All stool specimens were stored at −80°C immediately after processing. At study completion, stool samples were shipped to the central laboratory at University of Houston College of Pharmacy and were stored at −80°C until analysis.

### Outcomes

#### Safety assessment

Participants were monitored by obtaining vital signs and by direct observation during intravenous infusions for possible adverse events, including infusion reactions or hypersensitivity reactions. Participants were asked to report side effects at each visit and these were recorded by the research team. Adverse events were graded based on severity and were assessed for causality (related or not-related), duration and outcome.

#### Faecal pharmacokinetic analysis

Omadacycline stool concentrations were determined via LC-MS/MS as previously described.^17^ The lower limit of quantification was 0.1 ng/mL.

#### Microbiome analysis: proportional and quantitative changes in microbial taxa

Samples from baseline, during therapy (days 5–6), and end of therapy were used for this analysis. Stool DNA extraction was performed using the Eppendorf EPMotion 5075 liquid handling system following the Qiagen MagAttract PowerMicrobiome kit protocol (Qiagen, Hilden, Germany, catalogue no. 27500–4-EP). Extracted stool DNA was assessed for quantity and quality using the Qubit 4 Fluorometer (Thermo Fisher Scientific, Waltham, MA, USA). For quantitative microbiome analysis, the DNA was then diluted with polymerase chain (PCR)-grade water to 5 ng/µL and the DNA levels of bacterial groups were assessed using appropriate PCR primers/conditions as previously described.^10,18^ Primers used to quantify bacterial groups are shown in Table S1 (available as Supplementary data at *JAC* Online).^19–23^ Quantitative PCR (qPCR) was performed using the QuantStudio 5 (Applied Biosystems, Waltham, MA, USA).^16^ Threshold cycle values, expressed in copies per nanogram of DNA, were determined using a standard curve. The *R*^2^ values for standard curves and copies per gram of stool were calculated based on the initial DNA concentrations and weight of the stool aliquots. To assess relative microbiome abundance, the V4 region of the 16S ribosomal RNA gene was amplified using the dual indexing sequencing strategy as previously described.^24^ Sequencing was performed using the Illumina MiSeq (Illumina, San Diego, CA, USA).^16^

#### Bile acid analysis

Stool samples collected from baseline, end of IV therapy (day 5), and end of oral therapy (day 10) were used for this analysis. Standards for primary bile acids cholic acid (CA), chenodeoxycholic acid (CDCA), glycocholic acid (GCA), taurocholic acid (TCA), glycochenodeoxycholic acid (GCDCA) and taurochenodeoxycholic acid (TCDCA), and secondary bile acids lithocholic acid (LCA), deoxycholic acid (DCA), ursodeoxycholic acid (UDCA), alloisolithocholic acid (AILCA), glycolithocholic acid (GLCA), taurolithocholic acid (TLCA), glycodeoxycholic acid (GDCA) and taurodeoxycholic acid (TDCA) were purchased from Sigma-Aldrich (St. Louis, MO, USA). Standards for butyric acid, isobutyric acid, 2-methylbutyric acid, acetic acid, propionic acid, formic acid, valeric acid, isovaleric acid and hexanoic acid were obtained from Millipore-Sigma (Burlington, MA, USA). Optima^™^ LC/MS-grade solvents including water, acetonitrile, and methanol, and LC/MS-grade formic acid were all purchased from Fisher Scientific (Waltham, MA, USA) for the short-chain fatty acid method. The derivatizing reagent, 1-(3-dimethylaminopropyl)-3-ethylcarbodiimide hydrochloride, and the quenching reagents, succinic acid and 2-mercaptoethanol, were purchased from Fisher Scientific. Unlabelled aniline (used in the synthesis of the unlabelled SCFA standards) and [^13^C_6_]-aniline (used in the synthesis of SCFA-based internal standard compounds) were purchased from Millipore-Sigma (Burlington, MA, USA). For bile acid extraction, samples were aliquoted with faecal weights ranging from 20 to 100 mg and solubilized in a volume of methanol equivalent to 10 µL per 1 mg of stool. The mixture was incubated overnight at 4°C, followed by ultrasonification for 5 min, and then centrifuged at 10 000 **g** for 3 min. The supernatant was transferred to a new tube and analysed via liquid chromatography-tandem mass spectrometry (LC-MS/MS) as previously described.^25^ The final concentrations of each bile acid were normalized by the corresponding stool sample weight.

### Statistical analysis

Safety data were summarized using descriptive statistics. Faecal PK and quantitative microbiome qPCR analyses of bacterial taxa changes were assessed using medians with interquartile ranges. All statistical analyses were performed using SAS version 9.4 (SAS Institute, Cary, NC, USA) or R Software (v.4.4.1; R Core Team 2021).^26^ Proportional changes in 16S rRNA microbiome data were analysed using vegan R package (v.2.6.6.1; Oksanen 2024) and visualized using ggplot2 R package (v.3.5.1; Wickham 2016). CLC Genomics Workbench version 25 (Qiagen) was used for metagenomic assembly and the creation of the abundance table. Baseline relative abundance changes of bacterial taxa were assessed by linear logistic regression models using broom R package (v.1.0.6; Robinson 2024). Bacterial taxa significantly associated with omadacycline administration, bile acid changes, and SCFA changes were assessed by MaAsLin2.^27^

## Results

Between March and July of 2023, eight healthy volunteers (four female) aged 30 ± 4 years and a mean body mass index of 27 ± 4 kg/m^2^ were recruited and completed both intravenous and oral omadacycline therapy, as well as follow-up visits. All subjects were white (25% Hispanic). During the study period, a total of 14 adverse events (AE) were reported, including nausea (seven) and injection site symptoms (four). All AEs were considered mild (12) or moderate (two) and all resolved by the end of the study. None of the AEs required discontinuation of the study drug. All subjects provided one stool sample daily.

### Faecal pharmacokinetic analysis

**Figure 1.**
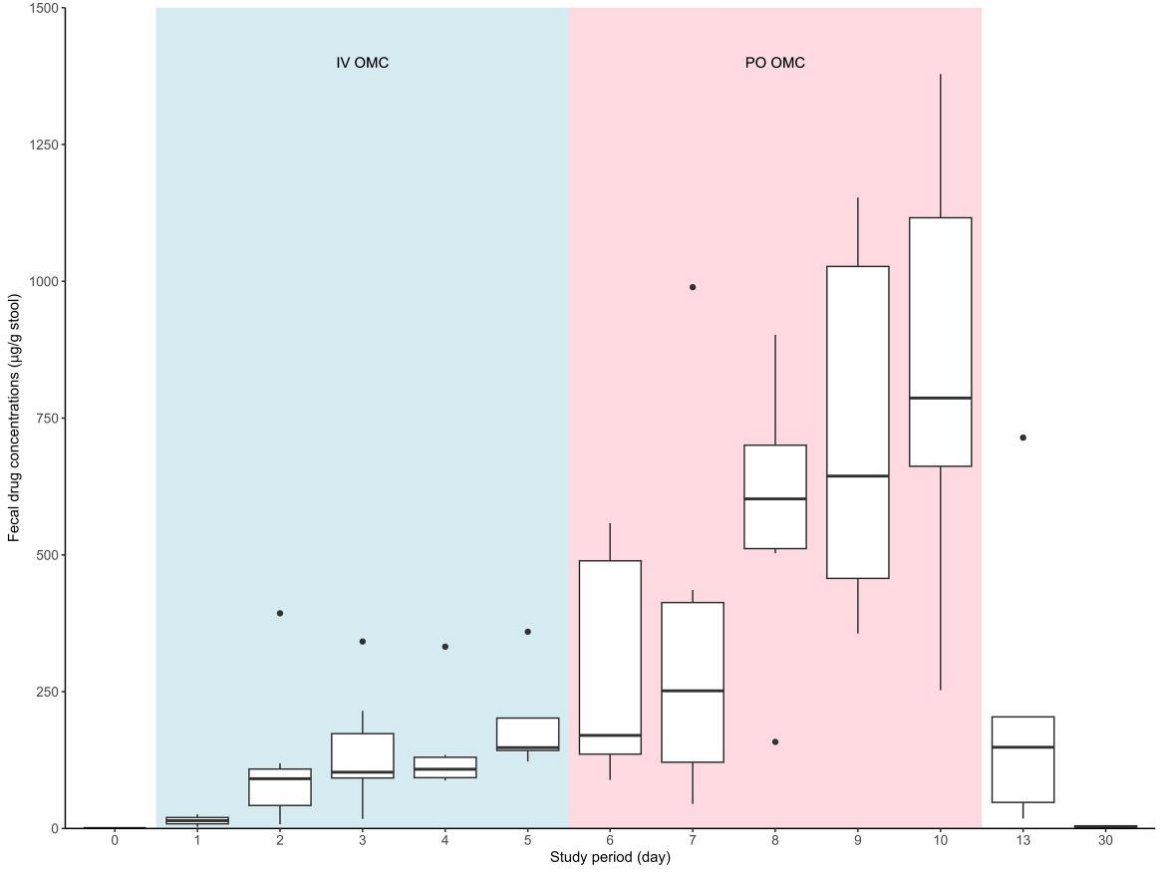
Faecal concentrations of omadacycline given intravenously (blue shading) or oral (pink shading).

A total of 83 faecal samples were analysed, all of which were categorized as type 4 or lower on Bristol Stool Chart (solid stool). Omadacycline was detected in all subjects after 48 hours of dosing and averaged 195 ± 97 µg/g (mean ± SD) by day 5 of omadacycline IV administration (Figure 1). Omadacycline concentrations increased rapidly after the start of oral therapy on day 6 with average concentrations of 854 ± 404 µg/g of stool by day 10. Omadacycline concentrations declined rapidly by the day 13 evaluation and were 5 µg/g stool or less at the day 30 follow-up visit.

### Quantitative and relative abundance microbiome analyses

**Figure 2.**
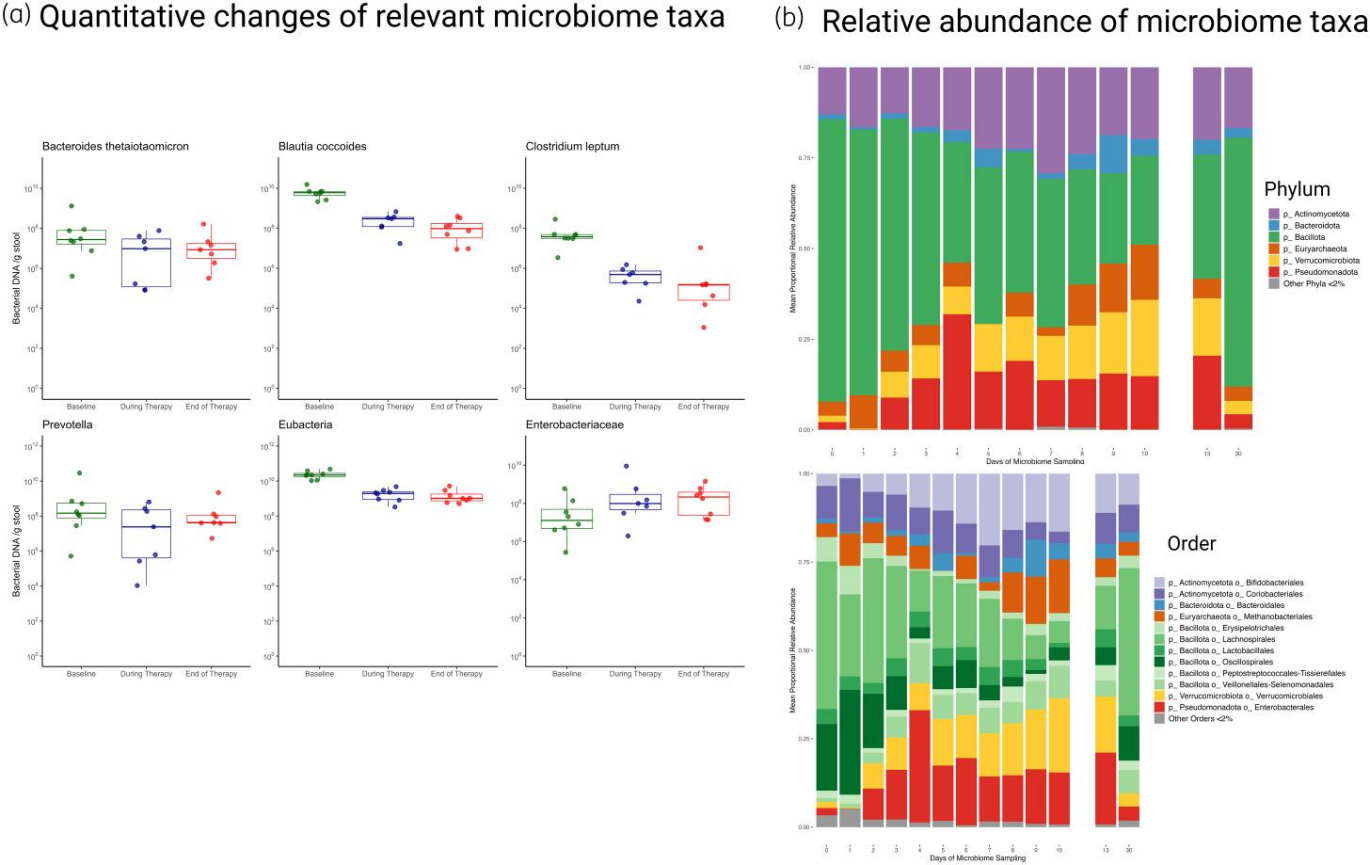
Quantitative changes of bacterial taxonomic groups (a) and microbiome relative abundance changes (b) from baseline during omadacycline intravenous and oral therapy.

For quantitative microbiome analyses, targeted qPCR analysis of samples collected from baseline, during therapy and end of therapy are shown in Figure 2. Specifically, *Blautia coccoides* and *Clostridium leptum* belonging to Bacillota phylum decreased over time but concentrations remained above 10^4–6^ bacterial DNA/g stool during intravenous and oral therapy. Other Bacteroidota (*B. thetaiotamicron*) and Pseudomonadota (Enterobacteriacaea) phyla remained consistent from baseline to intravenous and oral therapy time points.

Using MaAsLin2 and the 16S rRNA relative abundance table, changes from baseline after starting omadacycline therapy was significantly associated with increased relative proportions of the orders Bifidobacteriales, Verrucomicrobiales, Clostridiales, Enterobacterales and Burkholderiales and decreased proportions of several orders within Bacillota phylum, including Monoglobales, Lachnospirales, Oscillospirales, Erysipelotrichales and Christensenellales (Figure 2b). At the phylum level, a significant proportional decrease in Bacillota was observed (−5.1 ± 0.7%; *P* < 0.0001) and there were significant proportional increases in Actinomycetota and Verrucomicrobiota (+1.1 ± 0.3%; *P* < 0.001 and +1.7 ± 0.5%; *P* < 0.001, respectively). A non-significant proportional increase in Pseudomonadota phylum was noted (+0.9 ± 0.6%; *P* = 0.11). The decrease in Bacillota phylum was primarily driven by reductions in Lachnospiraceae (−3.3 ± 0.5%; *P* < 0.0001) and Ruminococcaceae (−1.5 ± 0.3%; *P* < 0.0001) families. The observed proportional increases in Actinomycetota and Verrucomicrobiota phyla were due to increases in Bifidobacteriaceae (+1.6 ± 0.2%; *P* < 0.0001) and Akkermansiaceae (+1.7 ± 0.5%; *P* < 0.001) families, respectively. Bacterial compositional changes in individual subjects are shown in Figure S1.

**Figure 3.**
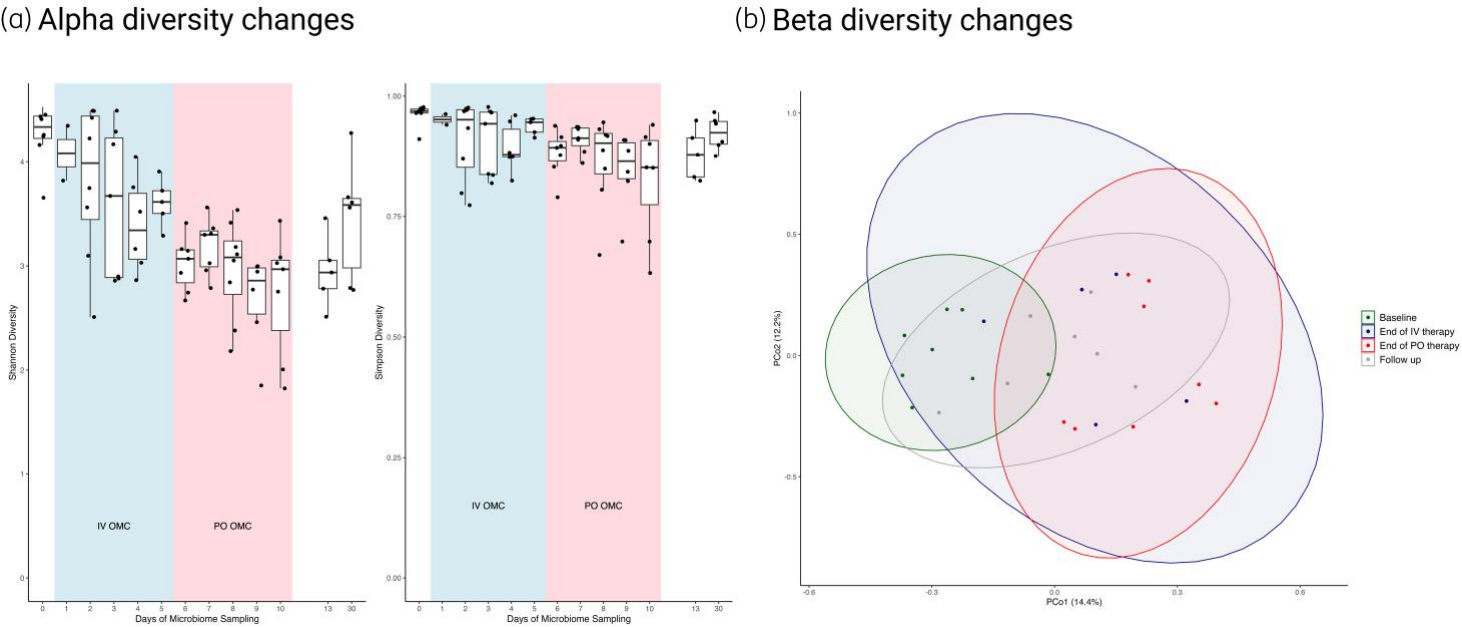
Alpha diversity changes (a) and beta diversity changes (b) from baseline in subjects given omadacycline intravenous (blue shading) followed by oral (pink shading) therapy.

Alpha diversity, measured by the Shannon index (Figure 3a), significantly decreased from baseline during IV therapy and continued to decrease after changing to oral therapy (*P* < 0.01) while alpha diversity changes measured by the inverse Simpson index showed no significant change. Beta diversity, assessed by the Bray–Curtis dissimilarity, showed a shift in bacterial composition from baseline over the study period, although there were considerable overlap among clusters across all timepoints (each ellipse represents a 95% confidence interval for each cluster) (Figure 3b). Samples collected at baseline were minimally similar to those collected at the end of oral therapy but follow-up samples overlapped with both clusters.

### Bile acid analysis

**Figure 4.**
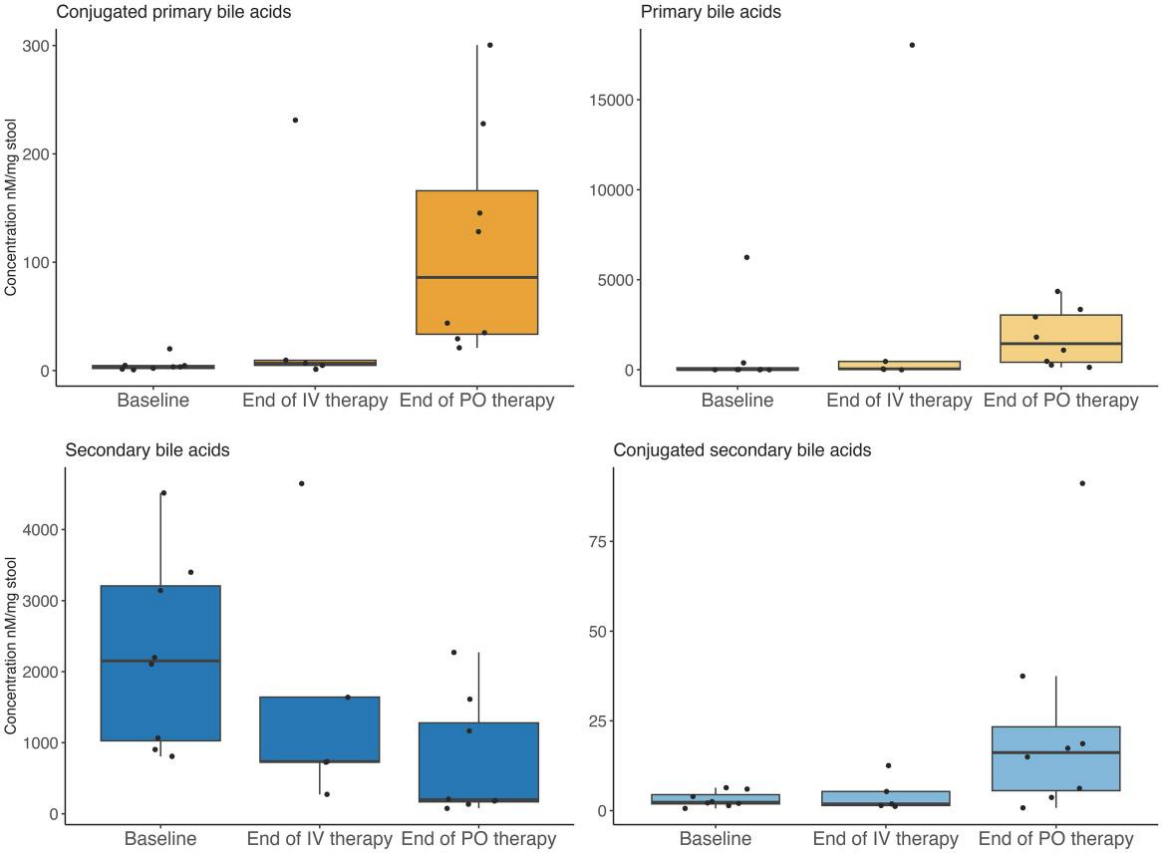
Summary of bile acid changes from baseline in subjects given omadacycline intravenous followed by oral therapy.

Faecal samples collected at baseline, end of IV therapy and end of oral therapy were used for bile acid analysis. Changes in conjugated and unconjugated primary and secondary bile acid concentrations are shown in Figure 4. Conjugated primary bile acids did not change appreciably from baseline during the 5 days of intravenous omadacycline but increased at the end of oral omadacycline therapy. There was no change in primary bile acids although primary bile acid concentrations were higher at the end of oral omadacycline therapy compared with the end of intravenous therapy. Secondary bile acids decreased at the end of intravenous omadacycline therapy and continued to decrease at the end of oral omadacycline therapy. Conjugated secondary bile acids were not significantly changed from baseline at the end of omadacycline intravenous therapy but numerically increased at the end of oral omadacycline therapy. The secondary bile acid, deoxycholic acid (DCA) did not change appreciably and ursodeoxycholate (UDCA) increased from baseline during oral and IV omadacycline therapy. Using MaAsLin2, bile acid changes were significantly associated with several orders within the Bacillota phylum. Decreased proportions of Christensenellales, Oscillospirales and Monoglobales orders were associated with increased primary bile acids while increased proportions of these orders were significantly associated with increased secondary bile acids. MaAsLin2 analysis indicated that increased proportions of Oscillospirales and Christensenellales orders were significantly associated with DCA.

## Discussion

CDI is a serious healthcare-associated infection in the USA and Europe, with metronidazole being the only intravenous treatment option recommended in treatment guidelines and specifically for fulminant disease. Metronidazole given orally was historically recommended as a first line treatment option but is no longer recommended due to declining clinical response rates and emerging antimicrobial resistance. Intravenous treatment options are needed for fulminant CDI cases but also for cases where oral therapy is not tolerated or when there is unreliable delivery of orally administered medication to the colon, e.g. ileus.

Omadacycline, a broad-spectrum antibiotic effective against aerobic and anaerobic bacteria including *C. difficile*, has demonstrated a low risk of inducing CDI in clinical trials.^13,15^ Given orally, omadacycline achieves high colonic concentrations and is less disruptive to the gut microbiome than oral vancomycin, preserving Actinomycetota phylum and key Bacillota groups.^16^ In this study, we demonstrated that a 5-day treatment with omadacycline given intravenously also rapidly achieves high stool omadacycline concentrations and is less disruptive than oral omadacycline or vancomycin, including the preservation of key microbial taxas responsible for metabolism of primary bile acids.^16^

Omadacycline stool concentrations increased after the change to oral omadacycline providing antimicrobial stewardship data to support intravenous to oral conversions for CDI treatment in the future. Further microbiome changes were observed after the conversion to oral therapy but preservation of bile acid homeostasis, evidenced by continued presence of secondary bile acids, was not significantly affected despite higher colonic omadacycline concentrations after the change to oral therapy. These results are consistent with our previous oral omadacycline study in which preservation of bile acid homeostasis was also observed.^15^

Omadacycline was detected in the stool at the first sampling time with concentrations reaching 195 µg/g of stool by day 5, well above the omadacycline MIC for *C. difficile*.^11^ A rat model study also demonstrated high concentrations of omadacycline within 30 minutes of infusion with ∼35% of the total dose being excreted in the faeces.^28^ Along with its microbiologic activity against *C. difficile*, the pharmacokinetic properties demonstrated in our study support the continued development IV omadacycline as an antibiotic option for treatment for CDI.

This study also adds considerably to our understanding of omadacycline effects on the gut microbiome. Microbiome effects with IV omadacycline were consistent with those previously demonstrated with oral omadacycline.^15^ However, IV omadacycline appears to be less disruptive than oral omadacycline or oral vancomycin, most likely due to lower omadacycline faecal concentrations with the IV formulation.^16^ Major microbiome changes demonstrated in this study include decreased Bacillota phylum, specifically Lachnospiraceae and Ruminococcaceae families, and proportional increases with Actinomycetota and Verrucomicrobiota phyla. These changes did not dramatically affect primary to secondary bile acid metabolism which has significant clinical implications as secondary bile acids, including deoxycholic acid, have been shown to inhibit germination of *C. difficile* spores.^29^ This may help explain the low propensity of omadacycline to cause CDI observed in previous clinical trials. The Oscillospirales order (belonging to the Bacillota phylum) was significantly associated with preservation of the secondary bile acid, deoxycholic acid.^30–32^ Deconjugation of CA or other primary bile acids to secondary bile acids requires bacterial transformation via 7-α dehydroxylation encoded by *bai* gene-carrying bacteria. Oscillospiraceae family are a taxa that has the *bai* gene, providing a mechanistic understanding for the findings from this study.^33^ Preservation of Christensenellales order bacteria and preservation of deoxycholic acid was another interesting finding. Presence of *Christensenella minuta* has been associated with human health benefits including lower body mass index, reduced serum lipids, decreased visceral adipose tissue and deconjugation of primary bile acids.^34^ Although not as well studied, *C. minuta* can metabolize primary bile acids to secondary bile acids via 3-O-acylation substitution, a structurally distinct bile acid. These results will require further validation.^35^ Last, Pseudomonodota was inversely associated with bile acids; however, this may simply be a biomarker of other reduced bacterial taxa rather than a direct effect of Pseudomonodota phylum.

This study has several limitations. We enrolled only a small number of healthy adults without any significant comorbidities. Microbiome effects of IV and oral omadacycline may be different in patients with chronic medical conditions. We were not able to perform metabolomic analysis on follow-up samples as sample volume was not adequate.^12^ A human gut models demonstrated omadacycline microbiome changes continued 7 to 10 days post antibiotic discontinuation; a time period we did not study. Although we demonstrated increased Bifidobacteria proportion using 16S rRNA analysis, a qPCR analysis to investigate quantitative differences was not undertaken. These results will need to be reconciled in the future. A comparison with fidaxomicin would also enable a direct comparison of microbiome effects. Finally, these study results will need to be validated in elderly patients with CDI.

In conclusion, intravenous omadacycline administration in healthy adults achieved high faecal concentrations while preserving key bacterial species in the gut. Our study showed the preservation of secondary bile acids following omadacycline administration, which has important implications and further supports Phase 2 studies directed towards the development of IV or oral omadacycline as a CDI-targeted antibiotic.

## Supplementary Material

dkaf278_Supplementary_Data

## Supplementary data

Figure S1 and Table S1 are available as Supplementary data at *JAC* Online.
