# Supplementary material for: Faecal pharmacokinetics, microbiome, and bile acid changes in healthy subjects given intravenous followed by oral omadacycline; a Phase 1 clinical trial
Source: J Antimicrob Chemother. 2025 Aug 12;80(10):2719–26. doi: 10.1093/jac/dkaf278 (PMC12494138; doi:10.1093/jac/dkaf278)

Supplemental Table 1. List of primers used to quantify bacterial groups

| **Bacterial Group (ref)** | **Gene** | **Sequences (5’-3’)** |
| --- | --- | --- |
| *Bacteroides*  *thetaiotaomicron* ^19^ | 16s rRNA | Forward_GAA GGT CCC CCA CAT TG  Reverse_CAA TCG GAG TTC TTC GTG |
| *Blautia coccoides ^20^* | 16s rRNA | Forward_GCT TCT TAG TCA RGT ACC G  Reverse_ACT CCT ACG GGA GGC AGC |
| *Clostridium leptum ^21^* | 16s rRNA | Forward_GCA CAA GCA GTG GAG T  Reverse_CTT CCT CCG TTT GTC AA |
| *Prevotella denticola ^20^* | 16s rRNA | Forward_GTA GGG GTT CTG AGA GGA  Reverse_AGC TGC CTT CGC AAT CGG |
| Eubacteria ^22^ | 16s rRNA | Forward_AGT TTG ATC CTG GCT CAG  Reverse_GWA TTA CCG CGG CKG CTG  Probe_FAM GCT GCC TCC CGT AGG AGT BHQ1 |
| Enterobateriaceae ^23^ | 16s rRNA | Forward_CAT TGA CGT TAC CCG CAG AAG AAG C  Reverse_CTC TAC GAG ACT CAA GCT TGC |

FAM=6-carboxyfluorescein; BHQ1=Black Hole Quencher 1

Supplemental Figure 1. Individual subject relative microbiome abundance changes from baseline


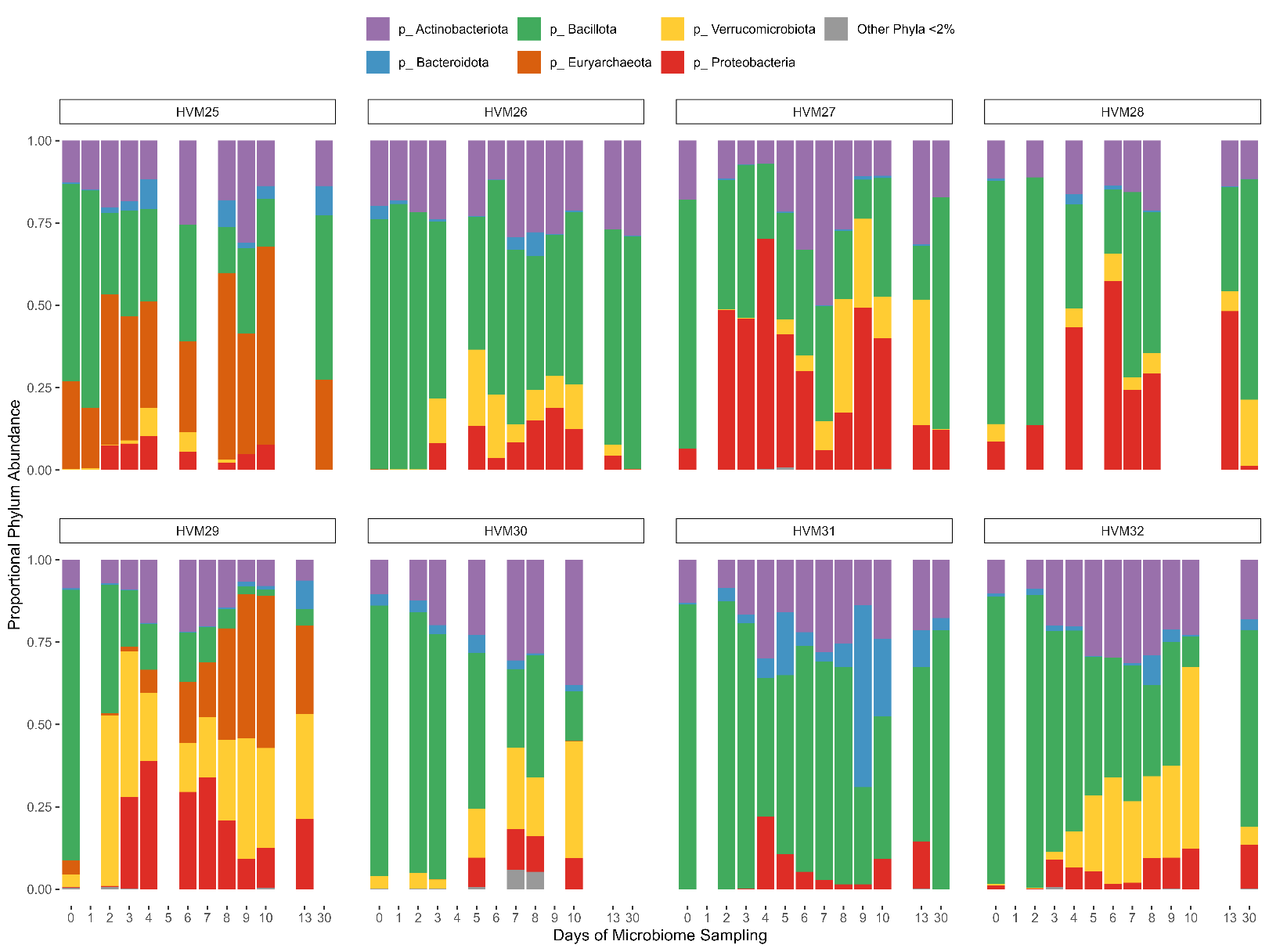

Supplement: dkaf278_Supplementary_Data [file dkaf278_supplementary_data.docx]
